# Programming living sensors for environment, health and biomanufacturing

**DOI:** 10.1111/1751-7915.13820

**Published:** 2021-05-07

**Authors:** Xinyi Wan, Behide Saltepe, Luyang Yu, Baojun Wang

**Affiliations:** ^1^ Centre for Synthetic and Systems Biology School of Biological Sciences University of Edinburgh Edinburgh EH9 3FF UK; ^2^ Hangzhou Innovation Center Zhejiang University Hangzhou 311200 China; ^3^ The Provincial International Science and Technology Cooperation Base for Engineering Biology International Campus Zhejiang University Haining 314400 China; ^4^ College of Life Sciences Zhejiang University Hangzhou 310058 China

## Abstract

Synthetic biology offers new tools and capabilities of engineering cells with desired functions for example as new biosensing platforms leveraging engineered microbes. In the last two decades, bacterial cells have been programmed to sense and respond to various input cues for versatile purposes including environmental monitoring, disease diagnosis and adaptive biomanufacturing. Despite demonstrated proof‐of‐concept success in the laboratory, the real‐world applications of microbial sensors have been restricted due to certain technical and societal limitations. Yet, most limitations can be addressed by new technological developments in synthetic biology such as circuit design, biocontainment and machine learning. Here, we summarize the latest advances in synthetic biology and discuss how they could accelerate the development, enhance the performance and address the present limitations of microbial sensors to facilitate their use in the field. We view that programmable living sensors are promising sensing platforms to achieve sustainable, affordable and easy‐to‐use on‐site detection in diverse settings.

## Graphical abstract

Synthetic biology offers new tools and capabilities of engineering microbes with defined functions as new biosensing platforms for environmental monitoring, disease diagnosis and adaptive biomanufacturing. Here, we summarize the latest advances in synthetic biology and discuss how they could accelerate the development, enhance the performance and address the present limitations of microbial sensors to facilitate their use in the field. We view that programmable living sensors are promising sensing platforms to achieve sustainable, affordable, and easy‐to‐use on‐site detection in diverse settings.

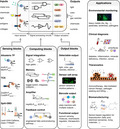

## Introduction

Microbial whole‐cell biosensors (WCBs) use the sensing functions of natural or genetically engineered microbes to achieve target detection, and are gaining increasing interests for many applications ranging from environmental monitoring to disease diagnosis in the rising era of synthetic biology. Compared to traditional biosensors, they have substantial advantages such as cost‐effective, easy‐to‐manufacture and biodegradable (van der Meer and Belkin, 2010; Saltepe *et al.,* 2018; Hicks *et al.,* 2020; Inda and Lu, 2020). Additionally, WCBs are renewable, providing sustainable economical solutions for food production (Rogers and Oldroyd, 2014), material synthesis (Choi and Lee, 2020), wastewater treatment and renewable energy generation (Cui *et al.,* 2019). Particularly for biomedical applications, they have potential to achieve non‐invasive *in situ* diagnosis and precision treatment (Inda and Lu, 2020). Here, we summarize the latest advances in synthetic biology and discuss how they could accelerate the development, enhance the performance and address the present limitations of living microbial sensors to facilitate their wide utilization in the field.

## Synthetic biology accelerates development of living sensors by providing standardized and modularized building blocks

**Fig. 1:**
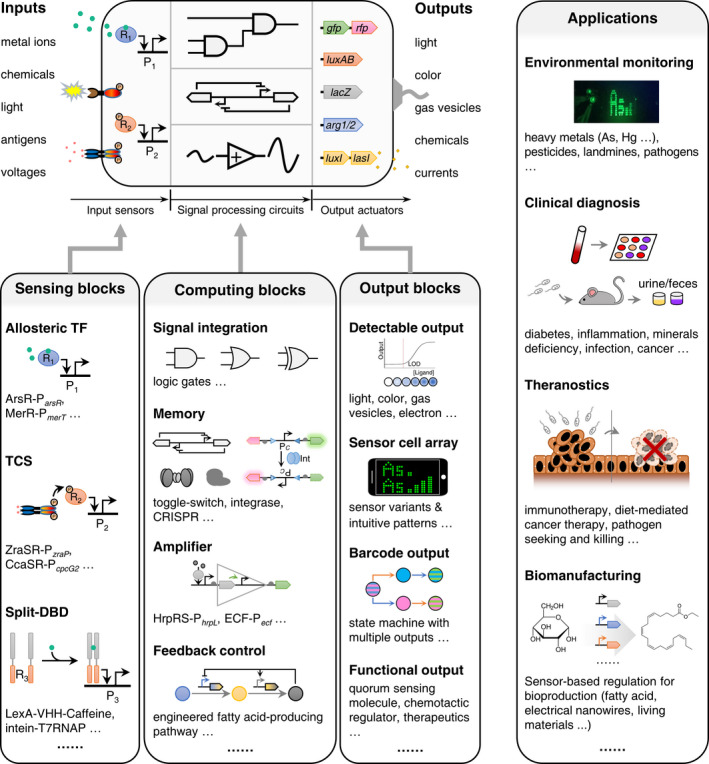
Programming living sensors for environment, health and biomanufacturing. Recent developments of synthetic biology provide numerous tools and modularized parts for programing cell‐based biosensors, including building blocks for the sensing module, the computing module and the output module. These blocks can be combined to achieve customized sensing and reporting specifications for diverse envisioned applications, such as environmental monitoring, clinical diagnosis, theranostics and biomanufacturing. R, receptor. P, promoter. *gfp*, gene encoding green fluorescent protein. *rfp*, gene encoding red fluorescent protein. *luxAB*, genes encoding bacterial luciferase for luminescent output. *lacZ*, gene coding β‐Galactosidase for colorimetric output. *arg*, acoustic reporter genes expressing gas vesicles that are detectable by ultrasound. *luxI* & *lasI*, genes encoding synthases for quorum sensing molecules. TF, transcriptional factor. ArsR, arsenic receptor. P*_arsR_*, ArsR’s cognate promoter. MerR, mercury receptor. P*_merT_*, MerR’s cognate promoter. TCS, two‐component system. ZraSR, zinc‐responsive histidine kinase and response regulator. P*_zraP_*, ZraR’s cognate promoter. CcaSR, green light‐responsive histidine kinase and response regulator. P*_cpcG2_*, CcaR’s cognate promoter. DBD, DNA‐binding domain. LexA, DNA damage or DNA replication inhibition‐responsive transcriptional repressor. VHH, a single‐domain antibody. Int, integrase. P*_C_*, constitutive promoter. HrpRS, hetero regulation motif in the *hrp* (hypersensitive response and pathogenicity) system of *Pseudomonas syringae*. P*_hrpL_*, HrpRS’s cognate promoter. ECF, extracytoplasmic function sigma factor. P*_ecf_*, ECF’s cognate promoter. LOD, limit of detection.

Synthetic biology offers scientists new tools to precisely manipulate cells for achieving bespoke tasks using engineered gene circuits of varying scales and complexity. Engineered WCBs generally comprise three main modules: (i) a sensing unit, (ii) a signal processing unit and (iii) an output unit (Fig. 1) (Wang and Buck, 2012). Most sensing units currently used are adapted from the natural cellular receptors such as ligand‐responsive transcription factor (TF)‐promoter pairs (Wang *et al.,* 2013a) or two‐component systems (TCSs) (Ravikumar *et al.,* 2012; Wang *et al.,* 2013a). Through optimal pathways, WCBs could be programmed to sense metal ions (Kim *et al.,* 2016; Wan *et al.,* 2019b), chemicals (Chong and Ching, 2016), metabolites (Liu *et al.,* 2015a), light (Fernandez‐Rodriguez *et al.,* 2017), electrical signals (Bhokisham *et al.,* 2020), temperature (Inda *et al.,* 2019) or even pressure (Fajardo‐Cavazos *et al.,* 2012). A simple and traditional WCB consists of a genetic reporter connected downstream a sensing unit to control the expression of a detectable output. For sensors incorporating more complex signal processing circuits, the sensing unit triggers more sophisticated actions before initiating reporter expression in order to enhance sensor’s performance or allow additional functions. Such circuits include logic gates (Wang *et al.,* 2011; Bonnet *et al.,* 2013), transcriptional amplifiers (Wang *et al.,* 2014; Wan *et al.,* 2019b), synthetic DNA sponges (Wan *et al.,* 2020), feedforward or feedback loops (Jia *et al.,* 2019), TF‐based (i.e. toggle switch) (Elowitz and Leibler, 2000; Gardner *et al.,* 2000) or RNA‐based switches (i.e. riboregulators) (Isaacs *et al.,* 2004; Green *et al.,* 2014), and memory circuits (Siuti *et al.,* 2013; Riglar *et al.,* 2017) (Fig. 1). Expression of any desired genes is referred as ‘reporter’ encoding detectable (Lopreside *et al.,* 2019; Del Valle *et al.,* 2021) or functional outputs (Hwang *et al.,* 2014; Din *et al.,* 2016; Chowdhury *et al.,* 2019) (Fig. 1). A noteworthy ‘reporter’ is to use DNA barcodes to record the changes in environment instead of continuous monitoring. Barcodes not only record input changes but also their orders providing useful information especially in disease monitoring (Roquet *et al.,* 2016; Sheth *et al.,* 2017; Tang and Liu, 2018). Further, instead of producing sensors with a single output, sensor cell arrays could be designed to display an easy‐to‐interpret output pattern corresponding to cognate input analyte levels without using sophisticated equipment (Wan *et al.,* 2019b; Kim *et al.,* 2020).

Inspired by mature engineering disciplines, synthetic biology adopts engineering principles (e.g. standardization, modularization and modelling) to facilitate complex circuit constructions particularly using ‘Lego‐like’ standardized building blocks (Endy, 2005). Although the blocks alone do not perform spectacular actions, they can generate bespoke coordinated functions when connected. Altogether, the advances in synthetic biology now allow both fine tuning the performance of existing WCBs and creating new biosensors with unique functionalities in a more predictable and rapid manner.

## Synthetic biology offers new toolkits to enhance performance of living sensors for applications in environment, health and biomanufacturing

Developments in industrialization have increased dissemination of pollutants and harmful substances which are threatening the environment and human health. Compared to traditional WCBs that use native stress response pathways to report general toxic environment (Kim *et al.,* 2005; Saltepe *et al.,* 2019), synthetic WCBs are able to detect specific pollutants such as heavy metals and metalloids (Wang *et al.,* 2013a; Wan *et al.,* 2019b), organic chemicals and pesticides (Chong and Ching, 2016), waterborne pathogens (Yong and Zhong, 2009) and explosives (Belkin *et al.,* 2017) (Fig. 1). Although many early‐stage WCBs are insufficient to meet real‐world detection requirements in limit of detection (LOD), selectivity and output amplitude, several gene circuit‐based optimization strategies have recently been developed to improve their sensing performance (Wan *et al.,* 2019a). In contrast to traditional optimization methods like random mutagenesis (Hakkila *et al.,* 2011; Chong and Ching, 2016), these synthetic biology‐enabled optimization tools are based on rational design, and therefore more predictable and rapid to achieve the desired sensing specifications (Wan *et al.,* 2019a). For example, simply integrating multiple inputs using genetic AND gates has been proven to be effective to increase WCBs’ selectivity (Wang *et al.,* 2013a; Wang and Buck, 2014), and rationally tuning the intracellular levels of the receptor TFs can quickly lower hence improve WCBs’ LOD (Wang *et al.,* 2015). In addition, a toggle switch (Wu *et al.,* 2009) and a post‐translational regulation device (Wan *et al.,* 2019b) have been designed to lower WCBs’ background expression and LOD. Further, amplification of the transduced sensor signal is another powerful strategy to further improve the sensor’s performance using strategies such as positive feedback loops (Jia *et al.,* 2019) or transcription amplifiers (Wang *et al.,* 2014; Wan *et al.,* 2019b).

Programming microbes for detecting health‐related biomarkers can lead to low‐cost point‐of‐care (POC) diagnostics as well as non‐invasive *in situ* diagnosis or theranostics (Riglar and Silver, 2018; Inda and Lu, 2020). They could report the disease both *ex vivo* (e.g. in urine or blood) (Courbet *et al.,* 2015) and in the body (e.g. in gut) (Riglar *et al.,* 2017). To date, myriad sensors have been engineered using bacteria to detect pathogens (Hwang *et al.,* 2014; Mao *et al.,* 2018), micronutrients (e.g. zinc) (Watstein and Styczynski, 2018) and disease biomarkers (Anderson *et al.,* 2006; Danino *et al.,* 2015; Riglar *et al.,* 2017; Isabella *et al.,* 2018) (Fig. 1). In some studies, specific therapeutics (e.g. cytotoxic agents) have been released *in situ* for precision treatment (Din *et al.,* 2016; Chowdhury *et al.,* 2019). Additionally, changes in disease progress could be recorded via memory circuits (e.g. toggle switches (Riglar *et al.,* 2017) or DNA recombinases (Courbet *et al.,* 2015)). Albeit remarkable progress has been reached to date, WCBs may face challenges regarding their performance in real‐world applications such as low signal‐to‐noise ratios or non‐specific results due to complex microenvironments. Nevertheless, the aforementioned transcriptional signal amplification circuits (Courbet *et al.,* 2015) and multiple signal integration using AND logic gates (Riglar and Silver, 2018) are viable solution to address these issues.

WCBs used in biomanufacturing have contributed to (i) real‐time monitoring of valuable compounds (e.g. nutraceuticals, pharmaceuticals and biofuels) (Liu *et al.,* 2015a; Rogers *et al.,* 2015), and (ii) stress monitoring in cells caused by nutrient (Brognaux *et al.,* 2013) and oxygen deficiency (Garcia *et al.,* 2009), or toxic intermediate production (Dahl *et al.,* 2013) during bioprocess. WCBs offer tremendous advantages such as facilitating rapid screening and selection of high‐producing strains among large mutant libraries, real‐time monitoring of metabolic flux, and detection of labile and low metabolites (Liu *et al.,* 2015a). Although many WCBs for metabolite monitoring are designed based on naturally occurring ligand‐responsive TFs and their cognate promoters, other approaches have recently been introduced in the field such as rational protein design to broaden the sensing capabilities of existing TFs (e.g. AraC) for metabolites with no existing receptor TFs (Tang and Cirino, 2011) and TCSs for extracellular metabolites (Ganesh *et al.,* 2015), or RNA switches to detect metabolites at lower concentrations (Fowler *et al.,* 2010). Moreover, dynamic sensor‐regulator circuits can be constructed in microbial cell factories to allow balancing metabolism and adaptively tuning product synthesis rate according to cell state change (Bradley and Wang, 2015; Liu *et al.,* 2015b) (Fig. 1).

## Synthetic biology provides novel strategies to overcome field‐deployable limitations of living sensors

Despite successful proof‐of‐concept demonstrations of WCBs in the laboratory, very few have made it into the market. Several barriers need to be overcome including inadequate number of sensory building blocks and insufficient knowledge of specific disease biomarkers, poor sensing performance, long‐term stability, risk of releasing genetically modified microorganisms (GMMs) and lack of practical experience in acceptance by professional stakeholders (Hicks *et al.,* 2020; Inda and Lu, 2020). Nevertheless, synthetic biology has contributed novel strategies to address these limitations to facilitate deployment of living sensors in the field.

One of the major limitations of circuit design is the insufficient number of well‐characterized genetic parts available in the toolkit of synthetic biology. Although attempts have been made to engineer new building blocks (e.g. rational protein engineering (Wang *et al.,* 2013b; Chang *et al.,* 2018)), they do not fit for all cases. Thus, to expand the existing library of genetic building blocks, synthetic biology could leverage advances from other fields such as machine learning. For instance, guided by deep learning, functions of RNA switches could be predicted resulting in shortened time required for their building and testing as well as reduced cost (Angenent‐Mari *et al.,* 2020; Valeri *et al.,* 2020). Deep learning‐derived prediction tools have also been developed to predict the transcription initiation frequency of synthetic bacterial promoters (Van Brempt *et al.,* 2020) and to predict TFs and their DNA‐binding domains from their protein sequences (Kim *et al.,* 2021). Additionally, machine learning has been introduced to increase the reliability for sensitive and specific detection of small molecules (Kim *et al.,* 2020; Saltepe *et al.,* 2021).

Most biosensors require calibration to generate reference response curves upon testing. Therefore, a portable, durable, inexpensive and user‐friendly platform for on‐site quantification is needed. Such devices have been utilized as prototypes for environmental contamination (Buffi *et al.,* 2011; Zhang *et al.,* 2020a) and health monitoring (Mimee *et al.,* 2018). Additionally, development of suitable platforms equipped with wireless connection will allow timely sensor data upload to a remote central database and easy monitoring (Liu *et al.,* 2020). Using electrochemical output could directly trigger relevant sensor device for monitoring and wireless reporting (Webster *et al.,* 2014); otherwise, an additional electronic device to convert the colorimetric or optical output signal into electrical signal would be required (Mimee *et al.,* 2018). Different approaches have been applied to keep biosensor cells alive and active for field deployment including freeze‐drying of cells (Bjerketorp *et al.,* 2006), encapsulating cells within polymers (Buffi *et al.,* 2011; Liu *et al.,* 2018; Wan *et al.,* 2019b) and continuous culture (Bjerketorp *et al.,* 2006; Wan *et al.,* 2019b). However, some aspects of these platforms are yet to be optimized such as the self‐renewability for long‐term monitoring and *in vivo* biotherapy, and the choice of materials suitable for long‐term storage (Liu *et al.,* 2017; Mimee *et al.,* 2018). Promising solutions may include adopting harsh condition‐resistant microbial chasses (Volpetti *et al.,* 2017; Guo *et al.,* 2018) or repurposing existing cell strains in the native sensing environment (Nejman *et al.,* 2020) for sensor development. Alternatively, a conventional cell chassis could be engineered or evolved to suit the target environment (Richard and Foster, 2003; Winkler *et al.,* 2014; Crook *et al.,* 2019).

Recent advances in synthetic biology allow harnessing the amazing sensing capabilities of microbes for versatile purposes, for example as wearable sensors for biomarker analysis in sweat to enable non‐invasive *in situ* real‐time health monitoring (Liu *et al.,* 2018; Chung *et al.,* 2019). However, biosafety concerns regarding the usage of GMMs remain an issue associated with their field applications including potential horizontal gene transfer and disruption of natural ecosystems (Dana *et al.,* 2012). Accordingly, different genetic containment strategies have been proposed to mitigate biosafety concerns such as replacing antibiotics resistance with auxotrophy (Hirota *et al.,* 2017) or toxin‐antitoxin systems (Wright *et al.,* 2015), incorporating conditional kill switches (Callura *et al.,* 2010; Chan *et al.,* 2016) and non‐canonical amino acid or xeno‐nucleic acid substitution (Pinheiro *et al.,* 2012; Fredens *et al.,* 2019). However, cells are prone to evolve and may escape from the engineered genetic safeguards. Hence, entrapment of cells in a biocompatible compartment minimizes the risk of accidental release of bacteria in the environment as well as protects them from hostile environment (Volpetti *et al.,* 2017; Liu *et al.,* 2018; Mimee *et al.,* 2018). Further, chromosome‐free bacterial chassis such as synthetic cells (e.g. minimal cells) constructed from bottom‐up approaches (Garamella *et al.,* 2016) and SimCells (Fan *et al.,* 2020) can be considered. Yet, a unique genome‐borne barcoding system for each chassis would allow handy tracing of any release and further minimizing safety concerns (de Lorenzo *et al.,* 2020).

In the last decade, cell‐free expression systems have become increasingly popular as a new sensor platform by avoiding safety concerns associated with using living cells. Cell‐free biosensors lend faster response, higher sensitivity and more tolerance to toxic samples (Silverman *et al.,* 2020; Zhang *et al.,* 2020b). Various cell‐free biosensors have been demonstrated to detect antibiotics (Jung *et al.,* 2020), pathogens (Pardee *et al.,* 2016; Takahashi *et al.,* 2018), toxic substances (Lopreside *et al.,* 2019; Jung *et al.,* 2020), etc. Moreover, cell‐free extracts comprising genetic sensors could be embedded on paper, providing a portable platform for easy‐to‐use and cost‐effective on‐site screening (Pardee *et al.,* 2016; Takahashi *et al.,* 2018), or in hydrogels acting as environment‐responsive biomaterials (Whitfield *et al.,* 2020).

## Outlook towards deploying living sensors in the field

Engineered living sensors have been pursued to fill the gaps left by conventional biosensing platforms by providing portable, easy‐to‐manufacture, cost‐effective and rapidly programmable platforms for on‐site detection. Despite demonstrated proof‐of‐concept success in the laboratory, few WCBs have made it into the market due to various restrictions. The latest advances in synthetic biology enable a rapid design–build–test cycle for sensor development and optimization to address the current limitations of WCBs. Yet, there are remaining challenges to be tackled both within and beyond the scope of technical developments.

Both environmental and health monitoring necessitate sensor cell exposure to complex samples and thus require complex signal processing circuits and even multiple input modules. Particularly for medical applications involving complex media compositions such as tumours, non‐specific localization of sensor cells prevents accurate diagnosis and biotherapy. To this end, engineering microbes for sensing and reporting at designated spatial locations will be crucial (Chien *et al.,* 2019). However, microbial sensors that support multiple spatiotemporal signals detection and integration have not been seen frequently due to technical challenges. Considering a single cell has a limited capacity in resources and large complex circuits tend to burden host cells, cell consortia comprising multiple communicating sensor strains may be used instead to facilitate multiplex detection (Wang *et al.,* 2013a; Khatun *et al.,* 2018).

Albeit cell‐free expression systems could address many issues facing WCBs, the genetic sensing circuits cannot always be transferred with the same or similar performance expected across the two platforms due to the fundamentally different biochemical environments. In addition, cell‐free systems have their own limitations, for example batch‐to‐batch variations and incompatible for continuous usage, to be addressed. *E. coli*‐based cell extract is the dominating cell‐free expression system at present. To meet different application needs, further work is expected to validate the use of other non‐model organism‐based cell‐free systems as alternative cell‐free sensing platforms (Zhang *et al.,* 2020b).

All in all, although living sensor platforms face certain restrictions, synthetic biology tools facilitate their adoption and use as promising alternative analytical devices to meet the real‐world detection requirements. To overcome remaining limitations, fundamental research is vital to identify new biomarkers and new candidate sensor elements as genetic building blocks. It will also provide necessary experimental data sets to feed and validate computational design platforms (e.g. machine learning or bioinformatics), with a goal to expand the standard and modular toolkits available for rapidly building synthetic biology‐enabled biosensors. In addition, multidisciplinary collaborations should be encouraged which will likely lead to novel practical solutions towards wide field deployment of living sensors. Considering the present biosensors for real‐world applications dominate in the healthcare sector, developing biosensors for environmental monitoring, biomanufacturing and other emerging scenarios will have significant space to grow and benefit diverse end users in the future.

This work was supported by the UK Research and Innovation Future Leaders Fellowship [MR/S018875/1], Leverhulme Trust grant [RPG‐2020‐241], US Office of Naval Research Global grant [N62909‐20‐1‐2036], Wellcome Trust Seed Awards in Science [202078/Z/16/Z] and Zhejiang University‐University of Edinburgh Joint Research Centre for Engineering Biology.

## Conflict of interest

None declared.
